# High co-expression of IL-34 and M-CSF correlates with tumor progression and poor survival in lung cancers

**DOI:** 10.1038/s41598-017-18796-8

**Published:** 2018-01-11

**Authors:** Muhammad Baghdadi, Hiraku Endo, Atsushi Takano, Kozo Ishikawa, Yosuke Kameda, Haruka Wada, Yohei Miyagi, Tomoyuki Yokose, Hiroyuki Ito, Haruhiko Nakayama, Yataro Daigo, Nao Suzuki, Ken-ichiro Seino

**Affiliations:** 1 Division of Immunobiology, Institute for Genetic Medicine, Hokkaido University, Kita-15 Nishi-7, Sapporo, 060-0815 Japan; 2 Department of Obstetrics and Gynecology, St. Marianna University School of Medicine, 2-16-1 Sugao, Miyamae-ku, Kawasaki City, Kanagawa 216–8512 Japan; 3 Department of Medical Oncology and Cancer Center, Shiga University of Medical Science, Otsu, 520-2192 Japan; 4 Center for Antibody and Vaccine, Research Hospital, Institute of Medical Science, University of Tokyo, Tokyo, 108-8639 Japan; 5 Molecular Pathology and Genetics Division, Kanagawa Cancer Center, Yokohama, 241-0815 Japan; 6 Department of Pathology, Kanagawa Cancer Center, Yokohama, 241-8515 Japan; 7 Department of Thoracic Surgery, Kanagawa Cancer Center, Yokohama, 241-8515 Japan

## Abstract

Despite recent advances in diagnosis and treatment of lung cancers, the 5-year survival rate remains unsatisfactory, which necessitates the identification of novel factors that associates with disease progression and malignant degree for improving diagnostic and therapeutic strategies. Recent progress in cancer immunology research has unveiled critical roles for colony stimulating factor 1 receptor (CSF1R) in multiple aspects of the tumor microenvironment. CSF1R is expressed on tumor-associated macrophages (TAMs), and mediates important pro-tumorigenic functions. CSF1R also provides critical autocrine signals that promote cancer cell survival and proliferation. Activation of CSF1R can be achieved by two independent ligands; macrophage colony-stimulating factor (M-CSF) and interleukin 34 (IL-34). Accordingly, the expression of these ligands in cancer is expected to result in poor prognosis. In this study, we show that IL-34 and M-CSF expression correlates with poor survival in a cohort of lung cancer patients. Importantly, high co-expression of IL-34 and M-CSF associates with the poorest survival compared to cancers that show weak or absent expression of the two ligands. Furthermore, high expression of IL-34 and M-CSF associates with advanced stages of lung cancers. Together, these results indicate a correlation between IL-34/M-CSF expression with poor survival and disease progression in lung cancer patients.

## Introduction

Lung cancer is the leading cause of cancer death and one of the most common cancers among both men and women worldwide^1^. In contrast to the steady increase in survival for most cancers, lung cancer still shows the poorest survival with less than 18% of 5-year relative survival, which results largely from poor detection and insufficient prediction of prognosis at early stages^1^. Obviously, an accurate assessment of prognosis is critical for an effective clinical decision and survival improvement.

With an aim to identify the molecular mechanisms that control the biological process of disease progression in cancer, several studies have focused on the genetic backgrounds of cancer cells and its relative impact on prognosis and clinical outcome of cancer therapy, such as RRM1, EGFR and KRAS mutations^2–6^. However, recent advances in cancer immunology research have unveiled a critical role for the interaction between cancer cells and immune cells at the tumor microenvironment (TME) in tumor progression and therapeutic resistance^7–9^. Thus, tumors that express critical immune-modulators are expected to be associated with high malignancy and thus related to poor prognosis. Indeed, patients with advanced stage cancers showed enhanced expression levels of several immune modulators including MIF, TNFα, IL-6, IL-8, IL-10, IL-18 and TGFβ^10^. Accordingly, accurate prediction of prognosis in cancer patients may require the assessment of such factors in addition to the genetic backgrounds of cancer cells.

Among several immune cells, tumor-associated macrophages (TAMs) consist the most abundant cell population in many tumors, which play crucial roles in multiple aspects of the TME, including tumor progression, invasion, metastasis and angiogenesis^11–13^. Importantly, TAMs infiltration has been considered as an independent poor prognostic factor in several cancers^7–9^. TAMs depend largely on CSF1R signaling for survival, proliferation and function, which can be achieved by two independent ligands; M-CSF and IL-34^14,15^. M-CSF and IL-34 share no sequence homology, but show comparable biological activities in myeloid cells^16,17^. Both cytokines correlate with tumor progression, metastasis, angiogenesis and therapeutic resistance^9^. It has been suggested that expression of IL-34 or M-CSF is accompanied with increased infiltration of M2-polarized TAMs that show enhanced pro-tumorigenic functions^18,19^. Based on these backgrounds, the expression of IL-34 and/or M-CSF at the TME may characterize tumors with enhanced aggression and has an impact on the patient’s survival. In this regard, previous reports have related M-CSF expression with poor survival in cancer patients^20,21^. However, IL-34 expression has not been evaluated in these studies, since it was discovered for the first time in 2008^22^. In this study, we analyze the expression of IL-34 and M-CSF in primary lung cancer tissues and its correlation with survival and tumor progression in a cohort of lung cancer patients, providing for the first time an evidence that show the association between IL-34 and M-CSF expression with disease progression and poor survival in lung cancer patients.

## Results

### IL-34 or M-CSF expression correlates with poor survival in lung cancer patients

**Figure 1:**
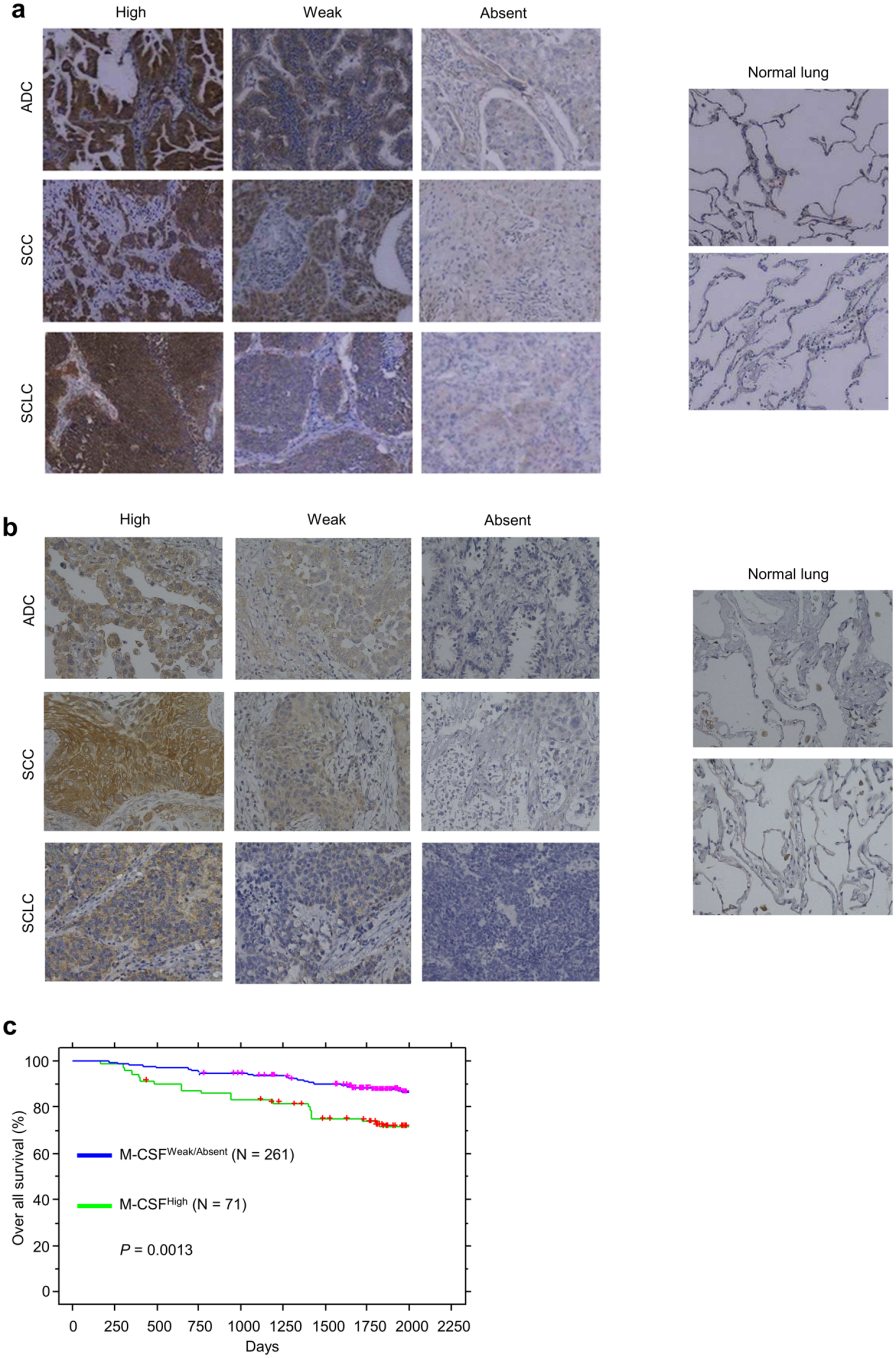
Correlation between IL-34 and M-CSF expression with poor survival in lung cancer patients. (**A**) and (**b**), Immunohistochemistry staining of IL-34 (**a**) or M-CSF (**b**) in primary lung cancer tissues from patients diagnosed with adenocarcinoma (ADC), squamous cell carcinoma (SCC) or small cell lung cancers (SCLC) compared to normal lung tissues. (**c**) Kaplan-Meier analysis showing overall survival in lung cancer patients according to M-CSF expression.

We have previously described a correlation between high expression of IL-34 and poor survival in a cohort of lung cancer patients (Fig. 1a)^19^. The clinicopathological characteristics of this cohort were described in detail in our previous report^19^. In this cohort, 45% of patients were Japanese women over 60 years without smoking history, and 77.4% of cases were diagnosed as non-small lung cancers (stage I) with 5 years of follow-up period^19^. Immunohistochemical staining of IL-34, M-CSF, CSF1R and CD163 was performed on lung cancer tissues obtained from patients by surgical resection. Antibodies specificity was confirmed before staining (Supplementary Fig. 1). Similar with IL-34, M-CSF expression was detected in lung adenocarcinomas (ADCs), squamous cell carcinomas (SCCs) and small cell lung cancers (SCLCs) with a variety among patients (Fig. 1b). In contrast, M-CSF was undetectable at protein level in normal lung tissues (Fig. 1b). Kaplan-Meier analysis of overall survival in lung cancer patients showed that high expression of M-CSF correlates with poor survival of lung cancer patients (Fig. 1c), in accordance with previous reports^20,21^.

### High co-expression of IL-34 and M-CSF correlates with the poorest survival in lung cancer patients

**Figure 2:**
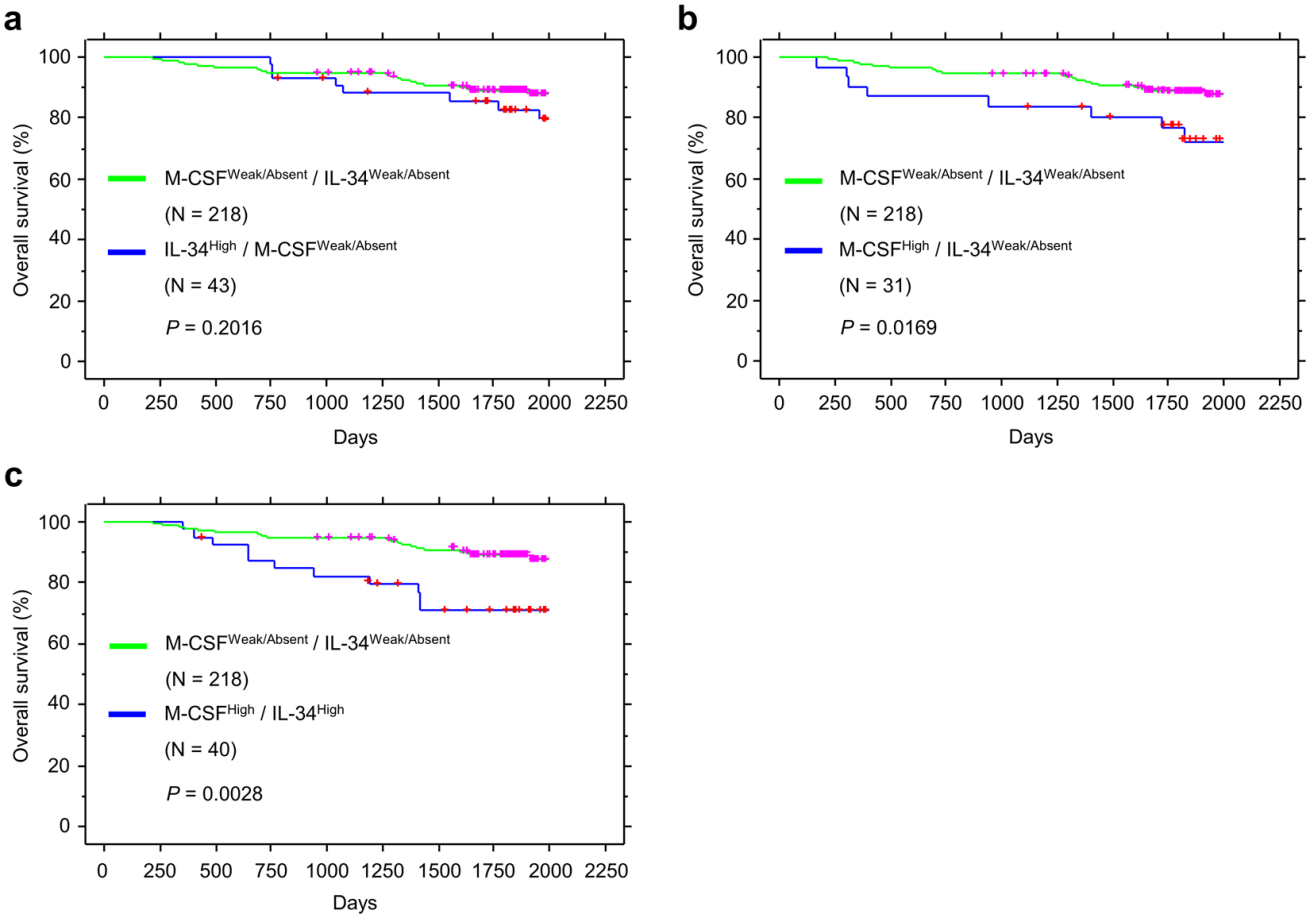
Correlation between M-CSF/IL-34 expression with poor survival in lung cancer patients. (**a**) Kaplan-Meier analysis showing overall survival in lung cancer patients that show M-CSF^Weak/Absent^/IL-34^Weak/absent^ expression compared to M-CSF^Weak/Absent^/IL-34^High^ group. (**b**) Kaplan-Meier analysis of overall survival in lung cancer patients that show M-CSF^Weak/Absent^/IL-34^Weak/absent^ expression compared to M-CSF^High^/IL-34^High^ group. (**c**) Kaplan-Meier analysis of overall survival in lung cancer patients that show M-CSF^Weak/Absent^/IL-34^Weak/absent^ expression compared to M-CSF^High^/IL-34^High^ group.

Next, we evaluated the association between IL-34 and M-CSF expression in lung cancers and the related impact on patients’ survival. Interestingly, 91% of cancer tissues that showed strong staining of IL-34 were accompanied with high (48%) or weak (43%) expression of M-CSF (Supplementary Table 1). Similarly, 77% of cancer tissues that showed strong staining of M-CSF were accompanied with high (56%) or weak (21%) expression of IL-34 (Supplementary Table 1). On the other hand, the absence of IL-34 staining in cancer tissues was frequently associated with the absence of M-CSF staining, and vice versa, (Supplementary Table 1) which may suggest a reciprocal relationship between the expression of IL-34 and M-CSF in lung cancers. Furthermore, we categorized patients in this cohort depending on the expression levels of M-CSF and IL-34 into 4 groups: 1) weak or absent expression of both M-CSF and IL-34, 2) High expression of IL-34 with weak or absent expression of M-CSF, 3) High expression of M-CSF with weak or absent expression of IL-34, 4) high expression of both M-CSF and IL-34. Kaplan-Meier analysis of overall survival in lung cancer patients based on this classification showed that high expression of M-CSF or IL-34 correlates with poor survival compared to groups that showed weak or absent expression of the two ligands (Fig. 2a,b). Importantly, patients with high expression of both M-CSF and IL-34 have the poorest survival compared to other groups (Fig. 2c). Together, these results suggest that high co-expression of both IL-34 and M-CSF correlates with poorer survival in lung cancer patients.

### CSF1R expression correlates with poor survival in lung cancers

**Figure 3:**
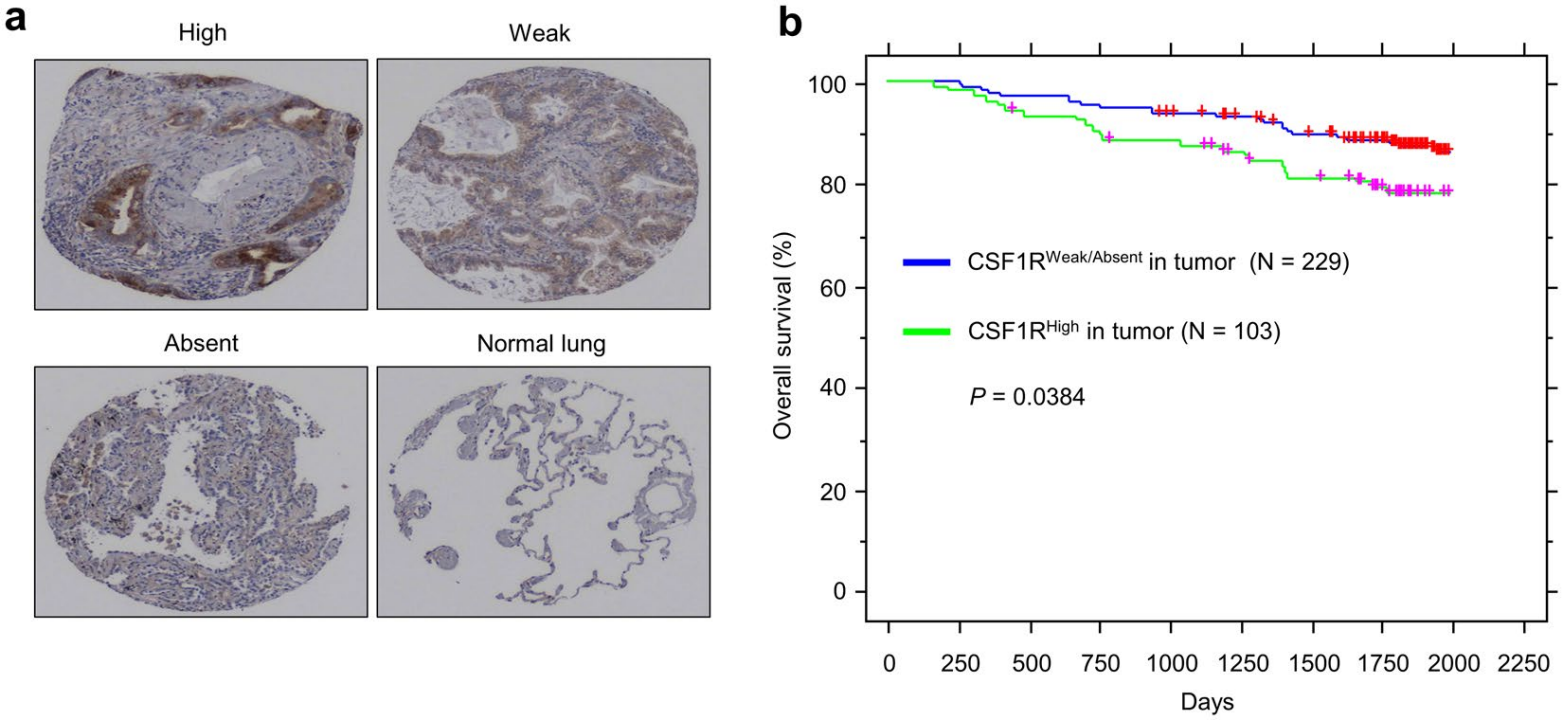
Correlation between CSF1R expression with poor survival in lung cancer patients. (**a**) Representative data of immunohistochemistry staining of CSF1R in primary lung cancer tissues compared to normal lung tissues. (**b**) Kaplan-Meier analysis showing overall survival in lung cancer patients according to CSF1R expression.

CSF1R is tyrosine-protein kinase that acts as a cell-surface receptor for M-CSF and IL-34^14–16^. CSF1R is expressed mainly in cells of the myeloid lineage and is a key regulator of macrophage differentiation^23^. However, several reports have showed that CSF1R expression can be also detected in other cells such as endothelial cells and importantly in cancer cell lines and primary cancer tissues^24,25^. Thus, we next evaluated the expression of CSF1R in lung cancer tissues. Similar with IL-34 and M-CSF, immunohistochemical staining showed that CSF1R is expressed in lung cancer tissues with a variety among patients (Fig. 3a). Again, high expression of CSF1R correlates with poor survival in lung cancer patients, similar with IL-34 and M-CSF expression (Fig. 3b).

### CD163 expression correlates with poor survival in lung cancers

**Figure 4:**
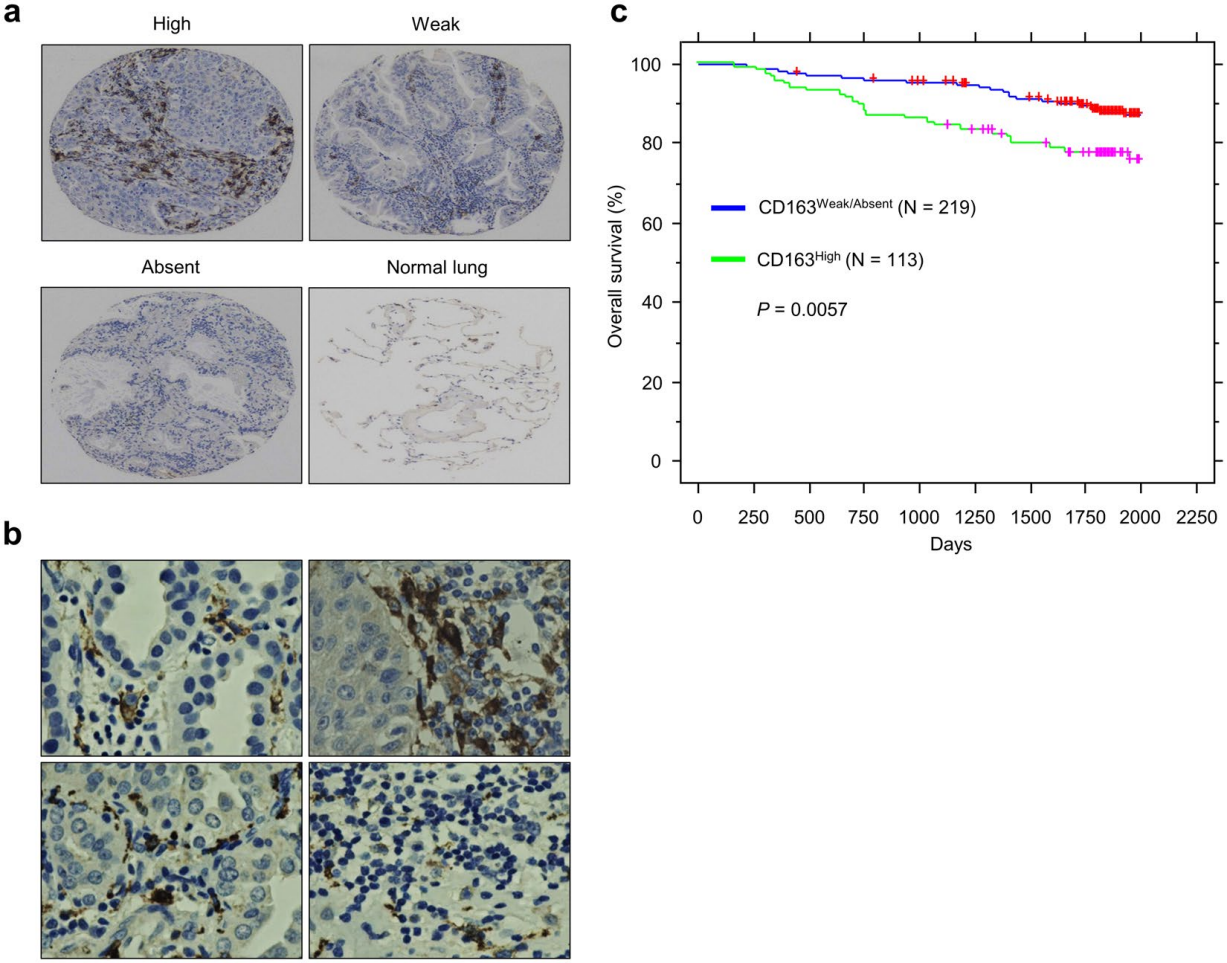
Correlation between CD163 expression with poor survival in lung cancer patients. (**a**) Representative data of immunohistochemistry staining of CD163 in primary lung cancer tissues compared to normal lung tissues. (**b**) High magnification images of CD163 staining in lung cancer tissues. (**c**) Kaplan-Meier analysis showing overall survival in lung cancer patients based on CD163 expression.

Accumulating evidence from clinical and experimental studies indicates that TAMs play critical roles in the promotion of tumor development, progression, metastasis and therapeutic resistance^26^. CD163 is a member of the scavenger family receptor, with high specificity for monocyte/macrophage lineage^27^, and has been considered as a specific marker to enumerate TAMs^28^. Thus, CD163 staining is expected to reflect the status of macrophage infiltration into tumors and predict poor prognosis in cancer patients^29^. Immunohistochemical staining showed that CD163 expression could be detected in lung cancer tissues with a variety among patients (Fig. 4a,b). Furthermore, Kaplan-Meier analysis of overall survival in this cohort of lung cancer patients showed that CD163 expression correlates with poor survival, in consistent with previous reports^29^ (Fig. 4c).

### IL-34 and M-CSF expression correlates with CD163 expression and poor survival in lung cancers

**Figure 5:**
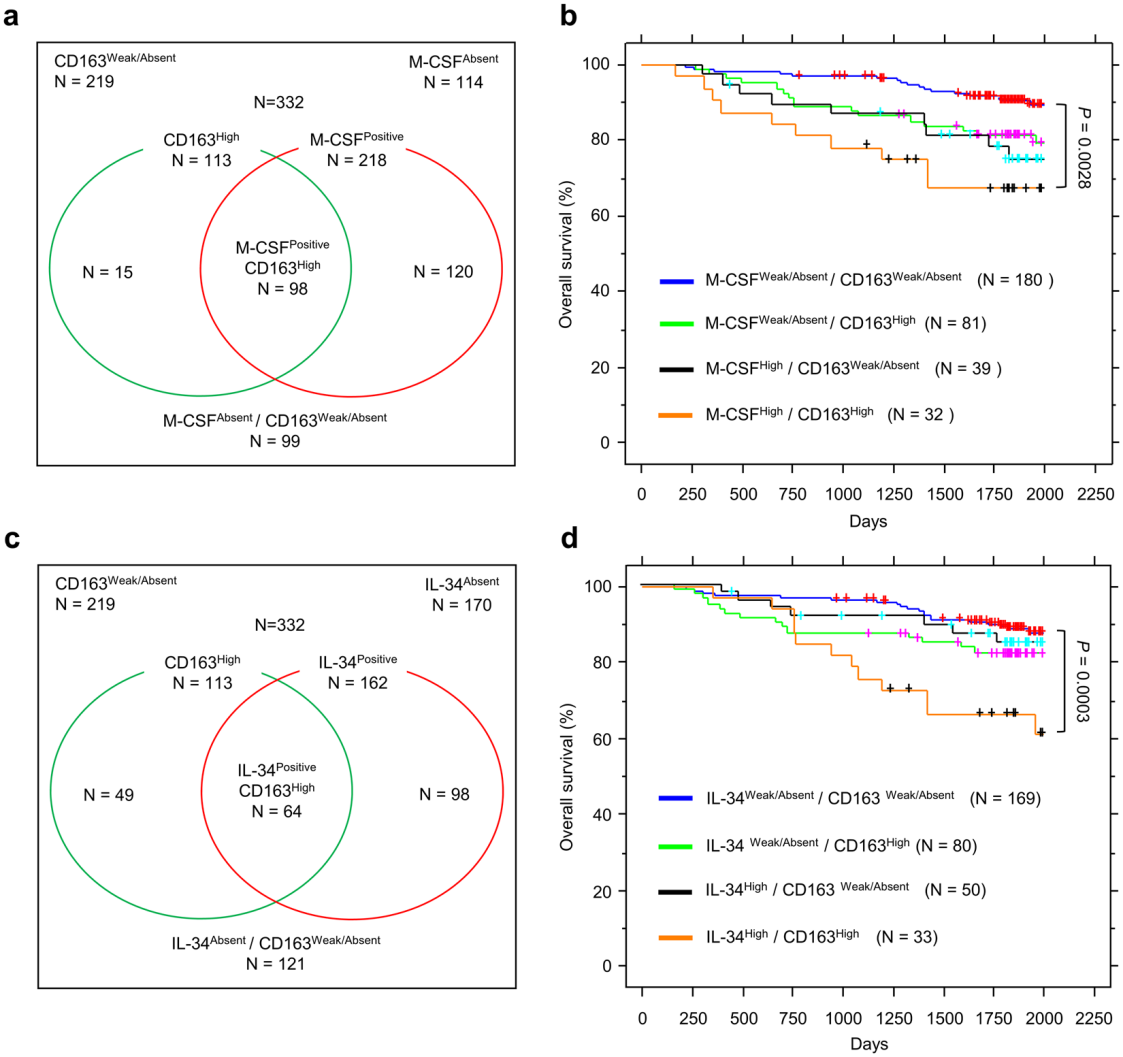
Correlation between M-CSF or IL-34 with CD163 expression in lung cancers. (**a**) Classification of lung cancer patients based on M-CSF and CD163 expression. M-CSF positive refers to samples that show high or weak expression of M-CSF. (**b**) Kaplan-Meier analysis showing overall survival in lung cancer patients based on M-CSF and CD163 expression. (**c**) Classification of lung cancer patients based on IL-34 and CD163 expression. IL-34 positive refers to samples that show high or weak expression of IL-34. (**d**) Kaplan-Meier analysis showing overall survival in lung cancer patients based on IL-34 and CD163 expression.

Next, we examined the relation between M-CSF or IL-34 with CD163 expression and its impact on survival in this cohort of lung cancer patients. High expression of M-CSF was frequently accompanied with high expression of CD163 (Fig. 5a; Supplementary Table 1), and the absence of CD163 staining was frequently associated with absent or weak staining of M-CSF (Fig. 5a; Supplementary Table 1). Kaplan-Meier analysis of overall survival in lung cancer patients based on M-CSF and CD163 expression showed that high expression of both M-CSF and CD163 in cancer tissues correlates with poor survival compared to other groups (Fig. 5b), consistent with previous reports^28^. Similarly, we evaluated the relation between IL-34 and CD163. As expected, 60% of cancer tissues that showed high expression of IL-34 were accompanied with high expression of CD163 (Fig. 5c; Supplementary Table 1), and the absence of CD163 staining was frequently associated with negative staining of IL-34 (Fig. 5c; Supplementary Table 1), which indeed suggests a correlation between IL-34 and CD163 expression in lung cancers. Kaplan-Meier analysis of overall survival in lung cancer patients based on IL-34 and CD163 expression showed that patients with high expression of both IL-34 and CD163 have the poorest survival compared to other groups (Fig. 5d). Collectively, these results indicate a correlation between high expression of IL-34, M-CSF, CSF1R and CD163 with poor survival, which was further confirmed in univariate analysis using Cox’s proportional hazards model (Table 1), although multivariate analysis in this model did not reach a statistical significance except for T and N factors (Table 1).

**Table 1:** Cox’s proportional hazards model analysis of prognostic factors in lung cancer patients.

Variables	Hazards ratio	95% CI	Unfavorable/Favorable	*P*-value
**Univariate analysis**				
High IL-34 expression	1.862	1.058–3.275	High/Weak and absent	0.031*
High M-CSF expression	2.445	1.390–4.303	High/Weak and absent	0.0019*
High CSF1R expression	1.774	1.023–3.077	High/Weak and absent	0.0411*
CD163 expression in TAM^#^	2.115	1.228–3.644	High/Weak and absent	0.0069*
Age (years)	1.624	0.910–2.899	≧ 65/< 65	0.101
Gender	1.539	0.875–2.706	Male/Female	0.1346
Histology	3.014	1.671–5.436	Non-ADC/ADC	0.0002*
T-factor	2.834	1.572–5.109	T2-3/T1	0.0005*
N-factor	3.67	2.097–6.422	N1-2/N0	<0.0001*
Smoking status	1.321	0.751–2.323	Ex or Current/Never	0.3342
**Multivariate analysis**				
High IL-34 expression	1.016	0.547–1.886	High/Weak and absent	0.9604
High M-CSF expression	1.492	0.799–2.785	High/Weak and absent	0.2089
High CSF1R expression	1.469	0.842–2.564	High/Weak and absent	0.1757
CD163 expression in TAM^#^	1.557	0.888–2.730	High/Weak and absent	0.1225
Histology	1.999	1.063–3.759	Non-ADC/ADC	0.0316*
T-factor	1.884	1.016–3.493	T2-3/T1	0.0445*
N-factor	2.911	1.617–5.241	N1-2/N0	0.0004*

*P < 0.05

^#^TAM: Tumor associated macrophage

ADC: Adenocarcinoma, Non-ADC includes Squamous cell carcinoma, Large cell carcinoma and others.

### IL-34 and M-CSF expression is enhanced in advanced stages of lung cancers

**Figure 6:**
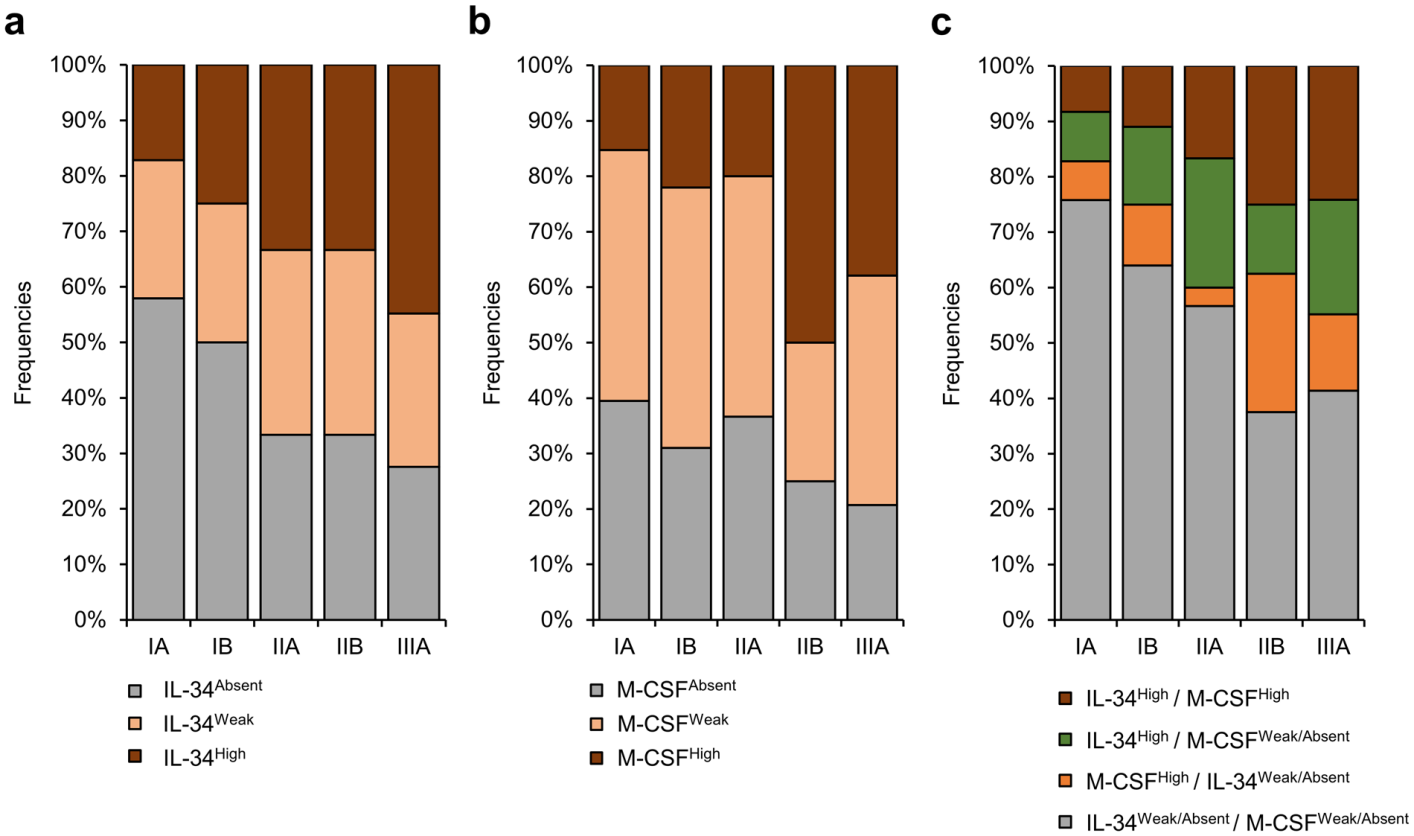
Association between IL-34/M-CSF expression with advanced stages in lung cancers. Bar graph analysis resembles the association between M-CSF or IL-34 expression with disease stages in lung cancer patients. Positivity rates of IL-34 (**a**), M-CSF (**b**) or both IL-34 and M-CSF (**c**) were calculated in each group according to disease stage.

Finally, we evaluated the expression level of IL-34 and M-CSF according to each stage in lung cancer patients (Supplementary Tables 2 and 3). By calculating positivity rates in each group, patients with high expression of IL-34 or M-CSF showed increased frequencies from stage IA (17.2%, 15.3%), stage IB (25%, 22%), stage IIA (40%, 20%), stage IIB (37.5%, 50%) to stage IIIA (44.8%, 37.9%) (Fig. 6a,b). Similarly, frequencies of patients with high co-expression of IL-34 and M-CSF associated with stages, starting from 8.3% at stage IA, 11% at stage IB, 16.7% at stage IIA, 25% at stage IIB, to 24.13% at stage IIIA (Fig. 6c). Statistically, the expression of IL-34 (Supplementary Table 4, P = 0.0004) or M-CSF (Supplementary Table 4, P = 0.0062) showed a tendency to be enhanced in stage II and III compared to stage I. Similarly, high co-expression of IL-34 and M-CSF was observed more frequently in stage II and IIIA compared to stage I (Supplementary Table 4, P = 0.0081). In a combined analysis, patients’ groups that showed high expression of IL-34 (Table 2, *P* = 0.0095), M-CSF (Table 2, *P* = 0.00829) or high co-expression of both IL-34 and M-CSF (Table 2, *P* = 0.0011) were associated with stage II and IIIA rather than stage I compared to other groups with weak or absent expression. Together, these findings indicate an association between IL-34 and M-CSF expression with stages in lung cancers, and thus may serve as progression parameters.

**Table 2:** Correlation between IL-34/M-CSF expression and disease stages in lung cancer patients.

Correlation between high IL-34 expression and disease stages				
	Total	IL-34^W/A^/M-CSF^W/A^	IL-34^High^/M-CSF^W/A^	*P*-value
	n = 261	n = 218	n = 43	
Stage				
IA	133	119	14	0.0095*^,#^
IB	78	64	14	
IIA	24	17	7	
IIB	8	6	2	
IIIA	18	12	6	
**Correlation between high M-CSF expression and disease stages**				
	**Total**	**IL-34** ^**W/A**^/**M-CSF** ^**W/A**^	**IL-34** ^**W/A**^/**M-CSF** ^**High**^	***P*** **-value**
	**n** **=** **249**	**n** **=** **218**	**n** **=** **31**	
Stage				
IA	130	119	11	0.00829*^,#^
IB	75	64	11	
IIA	18	17	1	
IIB	10	6	4	
IIIA	16	12	4	
**Correlation between high IL-34 and M-CSF co-expression and disease stages**				
	**Total**	**IL-34** ^**W/A**^/**M-CSF** ^**W/A**^	**IL-34** ^**High**^/**M-CSF** ^**High**^	***P*** **-value**
	**n** = **258**	**n** = **218**	**n** = **40**	
Stage				
IA	132	119	13	0.0011*^,#^
IB	75	64	11	
IIA	22	17	5	
IIB	10	6	4	
IIIA	19	12	7	

*P < 0.05 (Fisher’s exact test) ^#^stage I vs stage II-IIIA W/A: Weak or Absent.

## Discussion

In this paper, we describe for the first time the clinicopathological relevance of M-CSF and IL-34 expression with disease stages and poor survival in a cohort of lung cancer patients. Our data showed that single expression of M-CSF or IL-34 can be observed in primary lung cancer tissues and correlated with poor survival. High expression of both cytokines correlates with CD163 expression, which collectively correlates with poor survival. Additionally, high co-expression of M-CSF or IL-34 correlates with disease stages and the poorest survival compared to groups that showed weak or absent expression of the two ligands. Thus, evaluating the expression of both M-CSF and IL-34 may help to estimate disease progression and malignant degree in lung cancer patients.

In the cohort of lung cancer patients described in this study, IL-34 and M-CSF were naturally expressed in cancer tissues prior to any therapeutic procedures. Oncogenic mutations in cancer cells are frequently accompanied by activation of certain signaling pathways that induce the expression of a wide range of cytokines and chemokines, which in turn contribute to tumor progression and ultimately resistance to cancer therapy such as chemotherapy or tyrosine kinase inhibitors^30,31^. In this context, it is of great interest to identify oncogenic mutations that lead to IL-34 and M-CSF production by cancer cells and its impact on the tumor microenvironment, therapeutic resistance.

One remaining important issue is to unveil how can two ligands of the same receptor co-exist and exert their functions at the same microenvironment. The co-expression of both IL-34 and M-CSF has been previously observed in cancers such as giant cell tumors and malignant pleural mesothelioma^32–34^. In this study, we also observed a co-expression of M-CSF and IL-34 in a sub-population of lung cancer patients, which correlates with poorer prognosis. Under physiological conditions, M-CSF and IL-34 show tissue-restricted expression patterns with specific functions^14,15^. In *vitro*, both cytokines exhibit comparable biological functions in myeloid cells^14,15^. While expected to act as competitors, IL-34 and M-CSF can induce dual additive biological effects under certain conditions^17^. Additionally, IL-34 has the potential to interact with M-CSF to form a novel heterodimer that induce a specific activation pattern on CSF1R^17^. Accordingly, in tumors that naturally express both M-CSF and IL-34, or acquired the ability to produce both cytokines under certain therapeutic conditions, IL-34 has the possibility to act through interaction with M-CSF resulting in unique functions of CSF1R in both myeloid and cancer cells, which should be elucidated experimentally in further basic studies. Based on these backgrounds, co-expression of both IL-34 and M-CSF - naturally or induced under therapeutic conditions - may characterize malignancies with enhanced aggression and has an impact on the clinical outcome of cancer therapy. Indeed, our data shown in this study indicates an association between high expression of IL-34 and M-CSF in cancer tissues with disease stages and poor survival in lung cancer patients. In conclusion, IL-34 and M-CSF may help to predict poor survival and tumor progression in lung cancer patients, which should be further evaluated in other cohorts of lung cancer patients and various cancers in future studies.

## Materials and Methods

### Clinical Samples

Primary NSCLC tissues were collected from patients who had undergone surgical lobectomy or pneumonectomy at Kanagawa Cancer Center (Yokohama, Japan) after the acquirement of informed consent. A total of 332 resected tumor specimens were preserved at Kanagawa Cancer Center Biospecimen Center (KCC-BSC) and utilized for immunohistochemical analysis. All tumors were staged based on the pTNM pathologic classification of the UICC (International Union Against Cancer). All formalin-fixed samples of primary NSCLCs (Gender: 151 female and 181 male patients; Age: median age of 68 with a range of 35–90 years; Smoking history: 138 with no history of smoking, 192 ex- or current smokers and 2 unknown; Tissue type: 277 adenocarcinomas (ADC), 32 squamous cell carcinomas (SCC), 5 large cell carcinoma (LCC), and 18 other types of histological cancer; Stage: 157 pstage IA, 100 pstage IB, 30 pstage IIA, 16 pstage IIB, and 29 pstage IIIA cases) had been obtained earlier along with clinicopathologic data from KCC-BSC. A median follow-up period was 118.0 months for living patients (range, 8.0–138.9 months). The primary endpoint was overall survival as measured from the date of surgery to the time of death. Written informed consent was obtained from all patients, and the use of related clinical materials was approved by institutional ethics committees of Hokkaido University Hospital, Institute for Genetic Medicine and Kanagawa Cancer Center, and all experiments were performed in accordance with all guidelines and regulations indicated by these committees.

### TMA Construction

Tumor tissue microarrays were constructed with 332 formalin-fixed primary lung cancers, each of which had been obtained with an identical protocol to collect, fix, and preserve the tissues after resection. The tissue area for sampling was selected based on visual alignment with the corresponding hematoxylin and eosin (H&E)-stained section on a slide. Three to five tissue cores (diameter, 0.6 mm; depth, 3–4 mm) taken from a donor tumor block were placed into a recipient paraffin block with a tissue microarray (Beecher Instruments). A core of normal tissue was punched from each case, and 5-μm sections of the resulting microarray block were used for immunohistochemical analysis.

### Immunohistochemical analysis

To investigate the expression levels of IL-34, M-CSF, CSF1R and CD163 protein in clinical samples from lung cancer patients, tissue sections were stained the in the following manner. TMA slides were immersed in antigen retrieval solution (pH 9.0) (Nichirei, Tokyo, Japan) and boiled for 15 min in an autoclave. Endogenous peroxidase activity was blocked by incubation in 0.3% H_2_O_2_ in methanol for 15 min. After protein blocking (Catalog No. X0909, Abcam), TMA slides were incubated with a mouse anti-IL-34 antibody (Catalog No. ab101443), a rabbit anti-M-CSF antibody (Catalog No. ab52864, Abcam) in 1:100 dilution, a rabbit anti-CSF1R antibody (Catalog No. HPA012323, SIGMA) in 1:100 dilution or a mouse anti-CD163 antibody in 1:100 dilution (Catalog No. MCA1853, Bio-Rad) in Antibody Diluent (Catalog No. S0809, DakoCytomation) for 30 min at room temperature in a moist chamber. The sections were incubated with HRP-labeled polymer anti-mouse (Catalog No. K4007, DakoCytomation) or anti-rabbit IgG (Catalog No. K4002, DakoCytomation) as the secondary antibody for 30 min at room temperature in a moist chamber. Substrate-chromogen was added, and the specimens were counterstained with hematoxylin.

### Evaluation of Immunohistochemical Staining and Statistical analysis

Two independent investigators semiquantitatively assessed IL-34, M-CSF, CSF1R and CD163 positivity without prior knowledge of clinicopathologic data. Since the staining intensities of IL-34 and M-CSF were mostly homogenous in cytoplasm of cancer cells, they were semiquantitatively scored as high, weak or absent. As CSF1R staining was detected in cytoplasm and membrane, they were semiquantitatively scored as high, weak or absent. CD163 staining was mainly detected in cytoplasm of stromal macrophage. CD163^+^ macrophage infiltration in stroma was semiquantitatively scored as high, weak or absent, (high: many infiltrating CD163^+^ macrophages, weak: some infiltrating CD163^+^ macrophages and absent: no or few infiltrating CD163^+^ macrophages)^35^. If there is a discrepancy among them, a consensus was reached using simultaneous examination by two investigators.

### Statistics

Statistical analysis was done using the StatView software. Tumor-specific survival curves were calculated from the date of surgery to the time of death related to NSCLC or to the last follow-up observation. Kaplan–Meier curves were calculated for each relevant variable and for IL-34, M-CSF, CSF1R or CD163 expression; differences in survival times among patient subgroups were analyzed using the log-rank test.

## Electronic supplementary material

Supplementary Info
