# Supplementary material for: High co-expression of IL-34 and M-CSF correlates with tumor progression and poor survival in lung cancers
Source: Sci Rep. 2018 Jan 11;8:418. doi: 10.1038/s41598-017-18796-8 (PMC5765132; doi:10.1038/s41598-017-18796-8)
Supplement: Supplementary file 1 — Supplementary Info [file 41598_2017_18796_MOESM1_ESM.pdf]

Supplementary information

High co-expression of IL-34 and M-CSF correlates with tumor progression and poor survival in lung cancers

Muhammad Baghdadi, Hiraku Endo, Atsushi Takano, Kozo Ishikawa, Yosuke Kameda, Haruka Wada, Yohei Miyagi, Tomoyuki Yokose, Hiroyuki Ito, Haruhiko Nakayama, Yataro Daigo, Nao Suzuki, Ken-ichiro Seino

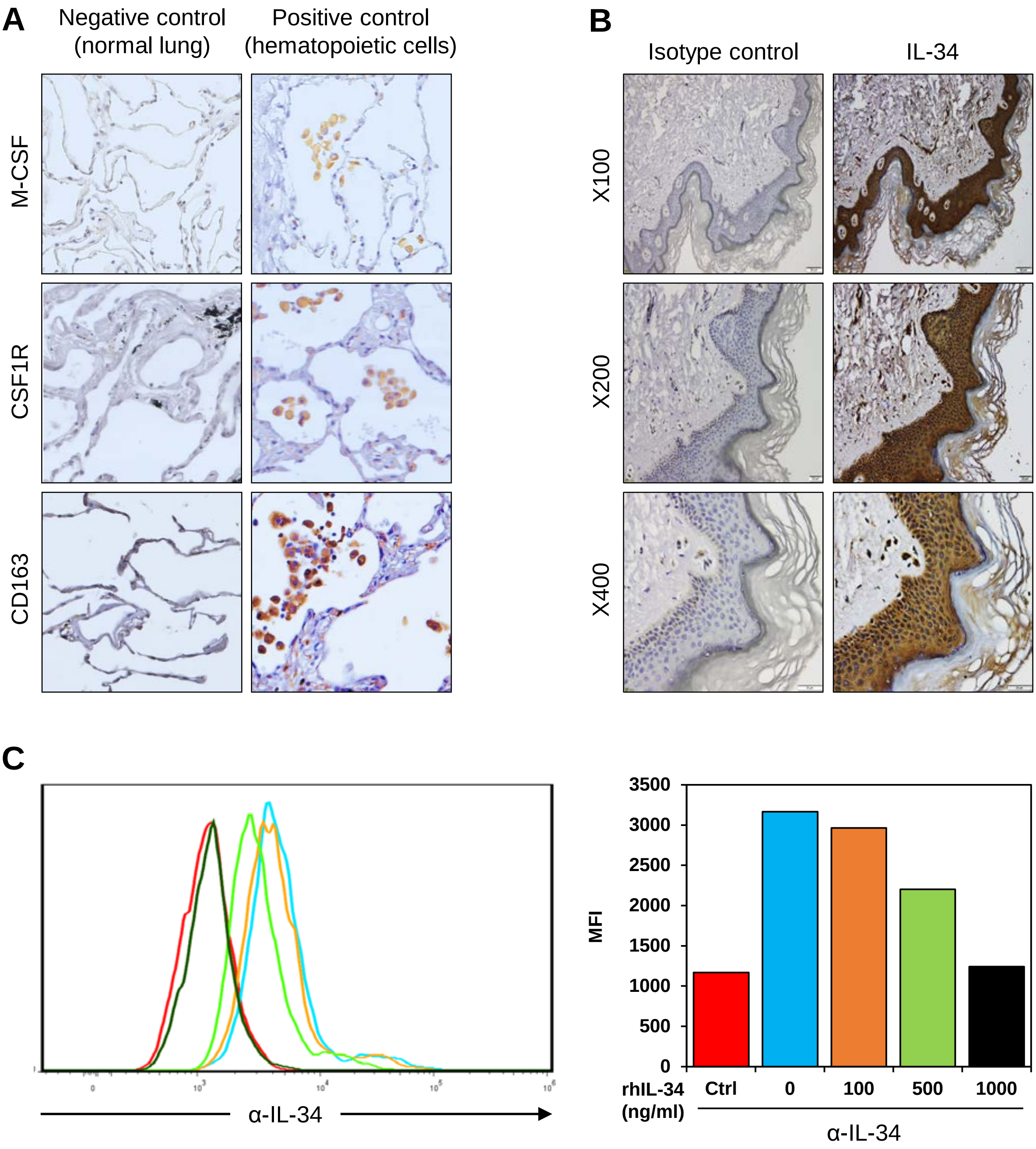

Supplementary figure 1

Confirmation of antibodies specificity. (**A**) Immunohistochemistry staining of M-CSF, CSF1R or CD163 is shown in hematopoietic cells (positive control) compare to normal lung (negative control). (**B**) Specific staining of IL-34 in keratinocytes of normal skin is shown by immunohistochemistry. (**C**) Intracellular staining of IL-34 in IL-34-producing A549 cells as analyzed by flowcytometry (left) and bar graph (right). Adding soluble antigen (recombinant human IL-34) results in decreased staining levels of IL-34 in a dose-dependent manner, indicating antibody specificity.

Supplementary table. 1

The relation between IL-34 / M-CSF / CD163 expression in lung cancer tissues

A. Relation between IL-34 and M-CSF expression in lung cancer tissues

	Total	IL-34 expression			*P*-value
		High	Weak	Absent	
	n = 332	n = 83	n = 79	n = 170	
M-CSF expression					
High	71	40	15	16	< 0.0001*
Weak	147	36	47	64	
Absent	114	7	17	90	

*P < 0.05 (Fisher's exact test)

B. Relation between M-CSF and CD163 expression in lung cancer tissues

	Total	M-CSF expression		*P*-value
		Positive	Negative	
	n = 332	n = 218	n = 114	
CD163 expression				
High	113	98	15	< 0.0001*
Weak or absent	219	120	99	

*P < 0.05 (Fisher's exact test)

C. Relation between IL-34 and CD163 expression in lung cancer tissues

	Total	IL-34 expression		*P*-value
		Positive	Negative	
	n = 332	n = 162	n = 170	
CD163 expression				
High	113	64	49	0.0488*
Weak or absent	219	98	121	

*P < 0.05 (Fisher's exact test)

Supplementary table. 2

Association between IL-34 expression and disease stages in lung cancer

		Total n = 332	High n = 83	Weak n = 79	Absent n = 170	*P*-value High vs. W/A
Gender						
	Male	181	48	44	89	0.5257
	Female	151	35	35	81	
Age (years)						
	< 65	143	38	32	73	0.6094
	65≧	189	45	47	97	
Histology						
	ADC	277	66	70	141	0.1148[#]
	SCC	32	10	7	15	
	LCC	5	2	1	2	
	Others	18	5	1	12	
Stage						
	IA	157	27	39	91	0.0004*, [##]
	IB	100	25	25	50	
	IIA	30	12	5	13	
	IIB	16	6	2	8	
	IIIA	29	13	8	8	
pT						
	T1	176	35	42	99	0.0305*
	T2-T3	156	48	37	71	
pN						
	N0	277	58	68	151	0.0003*
	N1-N2	55	25	11	19	
Smoking status						
	Never smoker	138	32	35	71	0.6064[###]
	Ex-smoker	157	42	40	75	
	Current smoker	35	8	4	23	
	Unknown	2	1	0	1	

*P < 0.05 (Fisher's exact test)

[#] ADC vs non-ADC

[##]stage I vs stage II-IIIA

[###]Never vs. Ex/Current smoker

Supplementary table. 3

Association between M-CSF expression and disease stages in lung cancer

		Total<br>n = 332	High<br>n = 71	Weak<br>n = 147	Absent<br>n = 114	*P*-value<br>High vs. W/A
Gender						
	Male	181	52	73	56	0.004*
	Female	151	19	74	58	
Age (years)						
	< 65	143	30	64	49	0.8934
	65≧	189	41	83	65	
Histology						
	ADC	277	44	126	107	<0.0001*,##
	SCC	32	17	12	3	
	LCC	5	3	2	0	
	Others	18	7	7	4	
Stage						
	IA	157	24	71	62	0.0062*,##
	IB	100	22	47	31	
	IIA	30	6	13	11	
	IIB	16	8	4	4	
	IIIA	29	11	12	6	
pT						
	T1	176	27	79	70	0.049*
	T2-T3	156	44	68	44	
pN						
	N0	277	53	124	100	0.0308*
	N1-N2	55	18	23	14	
Smoking status						
	Never smoker	138	19	64	55	0.0061###
	Ex-smoker	157	44	69	44	
	Current smoker	35	7	13	15	
	Unknown	2	1	0	1	

*P < 0.05 (Fisher's exact test)

ADC vs non-ADC

##stage I vs stage II-IIIA

###Never vs. Ex/Current smoker

Supplementary table. 4

Correlation between IL-34 and M-CSF expression and stages in lung cancer patients

A. Correlation between IL-34 expression and stages in lung cancer patients

		Total n = 332	High n = 83	Weak n = 79	Absent n = 170	*P*-value High vs. Weak/Absent
Stage						
	IA	157	27	39	91	
	IB	100	25	25	50	
	IIA	30	12	5	13	0.0004*,#
	IIB	16	6	2	8	
	IIIA	29	13	8	8	

*$P < 0.05$ (Fisher's exact test) #stage I vs stage II-IIIA

B. Correlation between M-CSF expression and stages in lung cancer patients

		Total n = 332	High n = 71	Weak n = 147	Absent n = 114	*P*-value High vs. Weak/Absent
Stage						
	IA	157	24	71	62	
	IB	100	22	47	31	
	IIA	30	6	13	11	0.0062*,#
	IIB	16	8	4	4	
	IIIA	29	11	12	6	

*$P < 0.05$ (Fisher's exact test) #stage I vs stage II-IIIA

C. Correlation between IL-34 / M-CSF expression and stages in lung cancer patients

		Total n = 332	IL-34 W/A M-CSF W/A n = 71	IL-34 High M-CSF W/A n = 147	IL-34 W/A M-CSF High n = 114	IL-34 High M-CSF High	*P*-value High vs. others
Stage							
	IA	157	119	14	11	13	
	IB	100	64	14	11	11	
	IIA	30	17	7	1	5	0.0081*,#
	IIB	16	6	2	4	4	
	IIIA	29	12	6	4	7	

*$P < 0.05$ (Fisher's exact test) #stage I vs stage II-IIIA W/A: Weak or Absent
